# Alexithymia and Cyberchondria: A Scoping Review with Implications for Healthcare Contexts

**DOI:** 10.3390/healthcare14111505

**Published:** 2026-05-28

**Authors:** Daniela Marchetti, Melissa D’Ettorre, Luigia Zito, Piero Porcelli

**Affiliations:** Department of Psychology, ‘G. d’Annunzio’ University of Chieti-Pescara, 66100 Chieti, Italy; melissa.dettorre@phd.unich.it (M.D.); zito.luigia1@gmail.com (L.Z.); piero.porcelli@unich.it (P.P.)

**Keywords:** cyberchondria, alexithymia, health-related internet use, health anxiety

## Abstract

Background: Cyberchondria is characterized by compulsive online health information seeking with additional psychological characteristics of behavioral addictions. Alexithymia, a transdiagnostic factor, is associated with difficulties in recognizing and differentiating emotions from bodily sensations. These characteristics may facilitate cyberchondria as a maladaptive strategy employed to cope with health anxiety. The present scoping review aims to examine the evidence regarding the association between alexithymia and cyberchondria. Methods: The scoping review was performed in accordance with the PRISMA-ScR guidelines. A comprehensive search of major databases (i.e., PubMed, Scopus, PsycINFO, and Web of Science) and grey literature sources (i.e., ProQuest and Google Scholar) was conducted. Data extraction was centered on the study’s design, the characteristics of the sample, the tools utilized, the primary findings, and other relevant variables. Results: A total of 139 records were identified from the databases, and four studies met the inclusion criteria. An additional study was selected from grey literature. The included studies involved different populations, including healthcare workers, university students, and patients with chronic conditions. Across these populations, a significant association between alexithymia and cyberchondria was consistently reported, considering both total scores and their respective dimensions. Furthermore, alexithymia mediated or moderated the relationship between other psychological factors (e.g., perceived stress, somatosensory amplification) and cyberchondria. Conclusions: The scoping review revealed limited but growing research indicating the potential influence of alexithymia on cyberchondria, with implications for clinical and healthcare contexts. The findings also highlighted gaps in the literature and the need for further research in this area.

## 1. Introduction

The use of the Internet for health-related information seeking has increased substantially over time, with a growing proportion of individuals relying on digital sources [1,2,3]. The accessibility, rapidity, cost-effectiveness, and anonymity of accessing medical information online may contribute to this trend [3]. Online searches have the potential to provide individuals with knowledge of disease mechanisms, etiologies, and prevention or treatment strategies, fostering a sense of empowerment and facilitating better-informed decisions about their health and healthcare [4,5]. On the other hand, health-related information seeking exposes individuals to inaccurate, unverified, or low-quality content, contributing to risk overestimation, increased self-diagnosis, heightened anxiety, overwhelming strain on medical resources, and potential disruptions in the doctor–patient relationship [6,7,8].

The repeated or excessive search for health-related information has been defined as cyberchondria, a pattern of compulsive and maladaptive online health information seeking [3,7] closely associated with health anxiety, distress, and functional impairment [9,10,11]. This phenomenon has been identified as a significant emerging clinical concern, as evidenced by numerous recent reviews that have summarized its definition [8,12], assessment methods [13], adverse consequences [11], and its impact on healthcare systems [8,14,15].

From a conceptual perspective, the phenomenon of cyberchondria has been interpreted within a variety of theoretical frameworks. Most authors discussed the excessive seeking of health-related information as a manifestation of health anxiety, defined as a psychological condition involving an excessive and persistent preoccupation with the fear of having or developing a serious medical illness [12,16,17]. Other researchers have posited that cyberchondria can be conceptualized within the framework of obsessive-compulsive behavior, which is characterized by compulsivity and the engagement in a safety behavior designed to alleviate somatic obsessions [10,18]. Moreover, the manifestation of cyberchondria has been proposed to align with the behavioral addiction framework, given its parallels with problematic internet use, including the loss of control over impulsive online activity, compulsive behavior, and the continuation of this behavior despite negative consequences, which leads to significant distress [19,20]. These perspectives reflect an ongoing theoretical debate and highlight several key characteristics of cyberchondria, including maladaptive health anxiety, compulsive reassurance-seeking, and features resembling behavioral addiction.

The repetitive online search for health-related information, a hallmark of cyberchondria, is closely associated with heightened vigilance toward bodily sensations and their potential misinterpretation. These processes underlie the tendency to somatize and report somatic symptoms more frequently [21]. The mechanisms in question face challenges in identifying and evaluating internal states. In light of this, individual differences in emotional awareness may hold particular relevance.

Alexithymia is originally conceptualized as a stable trait characterized by impairments in emotional awareness and processing [22,23], including difficulties in identifying and describing feelings, a tendency toward externally oriented thinking, and a limited imaginative capacity. Alexithymia has been consistently associated with somatic symptom reporting, health-related distress, and a wide range of psychopathological conditions of affective dysregulation, including addictive behaviors [24,25,26] and problematic internet use [27], suggesting that difficulties in emotional regulation may predispose individuals to maladaptive online behaviors. Alexithymia may increase vulnerability to cyberchondria through several mechanisms. Impaired emotional awareness and interoception may lead individuals to misinterpret physiological arousal as signs of physical illness, thereby amplifying health anxiety [27]. In this framework, online health information seeking may function as a maladaptive emotion regulation strategy aimed at reducing uncertainty and distress. However, repeated searches for symptoms may paradoxically exacerbate anxiety, reinforcing compulsive checking behaviors and symptom monitoring [28].

According to the established links between somatic focus, health anxiety, and cyberchondria [21], alexithymia may represent a critical dispositional factor influencing the onset and maintenance of these behaviors, with potential implications for healthcare settings. Research indicates that cyberchondria may interfere with patient–clinician interactions and increase healthcare utilization [9] and complicate clinical decision-making. Despite the growing empirical interest in both constructs, evidence regarding the relationship between alexithymia and cyberchondria remains fragmented and dispersed across different theoretical and methodological frameworks. A recent scoping review [29] summarized evidence on the effects of cyberchondria across various quality of life domains, including physical health, psychological health, level of independence, social relationships, environment, and behavior. Within the domain of psychological factors associated with cyberchondria, two studies reported a positive association between alexithymia and cyberchondria [29]. To date, no specific and comprehensive review has been conducted to examine the characteristics of the extant literature on the association between alexithymia and cyberchondria.

### Research Question

The present scoping review aims to examine and summarize the extant literature on the relationship between alexithymia and cyberchondria, as well as to identify gaps in the literature and highlight areas for future research. This scoping review addressed the following research questions:

1. What empirical evidence exists on the association between alexithymia and cyberchondria?
2. Which populations and settings have been studied in relation to the association between alexithymia and cyberchondria?
3. How are alexithymia and cyberchondria theorized and measured in research on this association?
4. Which additional factors have been investigated in relation to the association between alexithymia and cyberchondria?

## 2. Materials and Methods

The scoping review was conducted in accordance with the Preferred Reporting Items for Systematic Reviews and Meta-Analyses extension for Scoping Reviews (PRISMA-ScR) guidelines [30]. This scoping review’s protocol was pre-registered on the Open Science Framework on 26 February 2026 (https://osf.io/qhg32/).

### 2.1. Data Sources and Search Strategy

A comprehensive literature search was performed using the PubMed, Scopus, Web of Science, and PsycInfo electronic databases. The search was conducted without the application of any filters. The following keywords were utilized: ((cyberchondria OR cyberchondriasis OR cyberchondriac OR “health-related internet use” OR “Cyberchondria Severity Scale” OR “Cyberchondria Scale” OR “Short Cyberchondria Scale” OR “Brief Cyberchondria Scale” OR “Cyberchondria Tendency Scale”) AND (alexithymia OR alexithymic OR “Toronto Alexithymia Scale” OR “Bermond–Vorst Alexithymia Questionnaire” OR “Perth Alexithymia Questionnaire” OR “Toronto Structured Interview for Alexithymia”)). Furthermore, ProQuest and Google Scholar were also utilized to identify grey literature. For these sources, abbreviations of the assessment instruments were included in the search. Specifically, for Google Scholar, the initial 10 pages were searched following the platform’s default relevance ranking. Finally, the reference lists of all included articles were screened to identify additional pertinent studies that meet the inclusion criteria. The search encompassed publications up to 27 February 2026.

### 2.2. Eligibility Criteria

To be included in the scoping review, records had to meet the following criteria: (a) empirical studies reporting primary data, (b) peer-reviewed articles, theses/dissertations, or conference abstracts reporting original research, (c) written in English, Italian, French, or Spanish, (d) studies investigating the association between alexithymia and cyberchondria. Records were excluded if they: (a) did not report original empirical data, (b) were reviews, theoretical articles, or full texts that were inaccessible, (c) did not investigate the association between alexithymia and cyberchondria. No exclusion criteria were applied regarding the time frame, populations, or settings.

### 2.3. Study Selection

Duplicates resulting from the identification of relevant documents in the search sources were excluded. Subsequently, a preliminary evaluation of titles and abstracts was conducted by two independent reviewers to ascertain their alignment with the established eligibility criteria. Articles that were identified as potentially suitable for inclusion were retrieved and reviewed in full text to confirm their inclusion in the scoping review by two reviewers independently. Discrepancies were resolved through a consensus among the reviewers and the involvement of an additional reviewer.

### 2.4. Data Charting and Data Synthesis

The data were extracted from the papers included in the scoping review. The data items extracted included study information (e.g., authors’ names, publication year, region, etc.), study design (e.g., cross-sectional or cohort study), sample characteristics (e.g., sample size, age, gender), measures of alexithymia, measures of cyberchondria, results related to the association between alexithymia and cyberchondria, and all relevant variables that influence these relationships. The data extracted from the studies included were synthesized through the creation of tables, and a narrative summary of the evidence was provided based on the research questions.

## 3. Results

The results are organized into thematic sections aligned with the scoping review’s research questions, encompassing study selection and characteristics of the available evidence, populations and settings, measurement instruments used in the included studies, and the associations between alexithymia, cyberchondria, and related factors.

### 3.1. Evidence Selection and Characteristics of Included Studies

**Figure 1:**
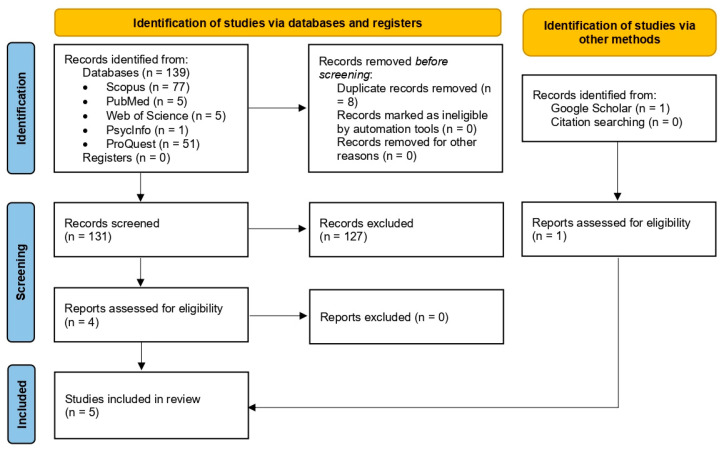
Modified PRISMA flow diagram.

The database search yielded 139 records. After removing duplicates, 131 records were screened based on title and abstract. This initial screening resulted in the identification of four studies that were assessed for full text and met the inclusion criteria. One additional study identified through the Google Scholar search also met the inclusion criteria and thus was incorporated into the review. In sum, five studies were included in the scoping review [31,32,33,34,35]. The study selection process is displayed in detail in a modified PRISMA flow diagram [36] (Figure 1).

The studies included in this scoping review were published recently, between 2022 and 2025. Three of the studies were conducted in China, and two were conducted in Turkey. The sample sizes of the included studies varied considerably, ranging from 190 [32] to 7617 [31] participants. All included studies employed observational designs with quantitative data, specifically cross-sectional designs. Table 1 presents the primary characteristics of the included studies and significant findings concerning the association between alexithymia and cyberchondria.

### 3.2. Populations and Settings

The studies included in the scoping review primarily focused on populations in healthcare contexts, including healthcare workers, patients, and university students. Two studies centered on nurses working in hospitals in China following the end of the zero-COVID policy. These nurses were at the forefront of addressing pandemic-related challenges, making them a target population for examining the relationship between alexithymia and cyberchondria in the context of pandemic-related fear and psychological distress [31,33]. Another study focused on the period of stress related to the COVID-19 pandemic on a large sample of college students in China [35]. Similarly, Sarpdaği et al. [34] recruited a sample of nursing university students, representing individuals preparing for healthcare professions.

Only one study [32] explored the association between alexithymia and cyberchondria in a clinical population, encompassing a sample of patients diagnosed with psoriasis in a hospital setting and a healthy control group, with a focus on a psychosomatic disease. The population and setting characteristics are delineated in Table 2.

### 3.3. Measures of Alexithymia and Cyberchondria

All the included studies assessed alexithymia using the Toronto Alexithymia Scale-20 (TAS-20) [37,38], a self-report questionnaire comprising 20 items designed to measure alexithymia in three dimensions, including Difficulty Identifying Feelings (DIF), Difficulty Describing Feelings (DDF), and Externally Oriented Thinking (EOT). Each item is evaluated using a five-point Likert scale, with higher total scores indicating higher levels of alexithymia. The validated Chinese [39] and Turkish [40] versions were utilized in accordance with the study populations.

Cyberchondria was assessed using the Cyberchondria Severity Scale (CSS) in its original 33-item version [41] in two studies and in its 12-item short version (CSS-12 [42]) in three studies. The CSS measures cyberchondria across five dimensions, namely compulsion (COM), distress (DIS), excessiveness (EXC), reassurance (REA), and lack of trust in medical professionals (MIS). The shorter CSS-12 assesses four dimensions comprising COM, EXC, DIS, and REA. Each item was evaluated using a five-point Likert scale, with higher total scores reflecting more severe cyberchondria. The authors employed different versions according to their populations, including the validated Turkish adaptations [43,44] and the measures previously used in Chinese samples [45].

### 3.4. Association Between Alexithymia, Cyberchondria, and Related Factors

The included studies reported significant associations between alexithymia and cyberchondria, although these relationships were investigated in different populations, using different analytical approaches, considering both total scores and dimensions of the variables of interest, and relating them to several psychological factors. The primary results from the included studies are presented in Table 1.

Two studies examined the association between alexithymia and cyberchondria in hospital nurses in China after the end of the zero-COVID policy, in relation to pandemic-related psychological factors. Fang et al. [31] found a significant and positive correlation between alexithymia and cyberchondria, showing also that pandemic fear was indirectly associated with cyberchondria through alexithymia and psychological distress. Concurrently, Li et al. [33] explored the complex relationships among pandemic fear, alexithymia, and cyberchondria using a multi-center network analysis. Their findings indicated the presence of a dense network characterized by predominantly positive connections, thereby underscoring the significant role of alexithymia. Specifically, EOT and DIF were negatively and positively associated with health-related behaviors, respectively. Two additional studies investigated the relationship between alexithymia and cyberchondria among student populations, exhibiting similarities to previous studies in terms of context and participant characteristics. Zhou et al. [35] found that both alexithymia and its dimensions were positively and significantly associated with cyberchondria and its components in a sample of Chinese college students during the period of the COVID-19 pandemic. Furthermore, alexithymia, specifically the DIF factor, was a significant predictor of higher levels of cyberchondria. In addition, the authors tested a moderation model, showing that alexithymia significantly moderated the association between perceived stress and cyberchondria [35]. In a different university sample of nurse students, Sarpdaği et al. [34] reported significant positive correlations between alexithymia and cyberchondria and their respective dimensions. In contrast to the findings of previous studies, the regression analysis also suggested a bidirectional association, indicating that cyberchondria and its COM dimension predict levels of alexithymia.

Finally, only one study examined the relationship between alexithymia and cyberchondria in a clinical sample of patients with psoriasis and healthy controls [32]. No significant differences were identified between patients and controls in alexithymia levels. Nevertheless, the control group exhibited a higher cyberchondria total score, COM and DIS subscales. Conversely, the psoriasis group presented higher levels of distrust of medical doctors (MIS subscale) and higher somatosensory amplification. Alexithymia was identified as a partial mediator of the association between somatosensory amplification and cyberchondria, exhibiting both significant direct and indirect effects on cyberchondria.

## 4. Discussion

### 4.1. Main Findings

This scoping review aimed to map the existing evidence on the relationship between alexithymia and cyberchondria. Overall, although based on a limited number of studies, the available evidence consistently suggests a positive association between alexithymia and cyberchondria, although the mechanisms underlying this relationship appear to vary depending on contextual and psychological factors. Across the studies, higher levels of alexithymia were associated with greater cyberchondria severity. The findings also suggest that specific components of alexithymia may play different roles in cyberchondria. In particular, the dimensions related to difficulty identifying and describing feelings were more consistently associated with cyberchondria severity, whereas the findings regarding the Externally Oriented Thinking (EOT) dimension were more heterogeneous. In addition, the reviewed studies suggest that alexithymia may interact with contextual stressors in shaping cyberchondria, including pandemic-related fear, perceived stress, and somatosensory amplification. The reviewed studies included diverse populations, such as healthcare professionals, university students, and clinical patients, although much of the available evidence derives from healthcare-related populations, especially nurses and nursing students. Despite the limited number of studies and populations examined, the assessment of the variables was consistent across studies. All studies employed the TAS-20 for alexithymia and the Cyberchondria Severity Scale (CSS or CSS-12) for cyberchondria, facilitating comparability across findings.

### 4.2. Interpretation and Theoretical Integration

These findings are consistent with the construct of cyberchondria as a maladaptive coping strategy for managing health-related uncertainty and anxiety [3,8]. However, given the correlational nature of the included studies, the following interpretations should be considered theoretical and hypothesis-generating rather than empirically established mechanisms. From a theoretical perspective, individuals with higher alexithymia may experience difficulties identifying and interpreting emotional states and bodily sensations [46], which could contribute to a greater reliance on external sources, such as online health information, when attempting to make sense of internal experiences. Within this theoretical framework, cyberchondria may be conceptualized as a digital form of reassurance-seeking behavior that maintains or exacerbates anxiety over time, creating a vicious circle [12]. From a theoretical perspective, the stronger associations observed for Difficulty Identifying Feelings (DIF) and Difficulty Describing Feelings (DDF) may reflect deficits in emotional awareness and emotional differentiation [47]. Individuals with difficulties identifying internal emotional states may struggle to distinguish between emotional arousal and physical symptoms. Consequently, emotional distress may be interpreted as a possible health problem, triggering further symptom monitoring and online health searches. This interpretation is consistent with research showing that alexithymia is associated with increased attention to bodily sensations and somatic misinterpretation [48]. In accordance with this perspective, one included study [32] revealed that alexithymia mediates the relationship between somatosensory amplification and cyberchondria, supporting the notion that difficulties in emotional awareness and regulation may amplify the impact of heightened bodily sensation perception on cyberchondria. By contrast, the findings regarding the EOT dimension were more controversial, with studies reporting both positive [34,35] and negative associations [33,35]. Previous literature has suggested that EOT may represent a cognitive style more closely related to deficits in imaginal activity, whereas DIF and DDF are more strongly linked to emotional dysregulation [46,49]. This may partly explain the inconsistent findings observed in the extant literature. However, the observed variability may also reflect methodological and psychometric factors. Prior research has indicated that the EOT dimension of the TAS-20 may exhibit lower internal consistency and less stable factor structure compared to other alexithymia dimensions [50]. In addition, differences across study populations, contextual factors, and analytical approaches, including the use of different cyberchondria dimensions and statistical models, may have contributed to the heterogeneity of the findings.

The reviewed studies also suggest that alexithymia may interact with contextual stressors in shaping cyberchondria. For instance, one study found that alexithymia moderated the relationship between stress and cyberchondria, while another showed that alexithymia mediated the relationship between pandemic-related fear and cyberchondria. These findings are consistent with broader models suggesting that alexithymia is associated with difficulties in emotion regulation [47] and increasing vulnerability to external stress and the use of maladaptive coping strategies under conditions of uncertainty and distress [51]. The predominance of healthcare-related populations may also have theoretical relevance. The high exposure of healthcare workers to medical information may result in increased symptom monitoring and perceived health risks, as greater medical knowledge does not necessarily reduce health anxiety [52]. During the pandemic, frontline healthcare workers experienced significant psychological distress and uncertainty, which may have contributed to higher levels of cyberchondria [31]. Furthermore, although alexithymia shows a well-established association with chronic and functional medical conditions [53,54], empirical evidence examining its relationship with cyberchondria in clinical populations remains very limited, with only one study involving patients with psoriasis [32]. Taken together, these preliminary findings suggest that alexithymia may represent a potential transdiagnostic vulnerability factor contributing to maladaptive health-related online behaviors.

From a clinical perspective, interventions aimed at improving emotional awareness, emotional labeling, and emotion regulation skills may help reduce the tendency to engage in excessive online health searches. Approaches targeting interoceptive awareness and emotional differentiation may be particularly relevant, as they may help individuals better interpret bodily sensations and reduce the misattribution of emotional arousal to physical illness. Although the findings across the included studies consistently suggest a positive association between alexithymia and cyberchondria, the current state of evidence should be interpreted within the context of an emerging and still underdeveloped research field.

### 4.3. Limitations and Future Directions

Despite the emerging evidence, the limitations of the included studies should be addressed. Firstly, longitudinal design was not employed in any of the included studies. Consequently, the cross-sectional design of all the studies precludes causal conclusions and temporal inferences. Secondly, the studies employed exclusively self-report instruments, which may introduce reporting and social desirability biases, potentially affecting the validity of the findings. Thirdly, the samples were predominantly convenience-based and consisted largely of university students and healthcare professionals in hospital settings. This limitation reduces the representativeness of the findings and their generalizability to broader populations. Differences in educational levels may also have influenced cyberchondria scores. Furthermore, the studies were primarily concentrated in China and Turkey, often in pandemic-related contexts, which limits the possibility of considering cultural or regional variations and differences between healthcare systems in non-pandemic contexts. Concurrently, the conceptualization of cyberchondria remains a subject of debate within the extant literature, further limiting the interpretability of the findings. The phenomenon of cyberchondria is frequently conceptualized within the framework of behavioral addiction. However, none of the included studies examined its comorbidity with problematic internet use or other behavioral addictions. This finding represents an additional limitation, as other factors may influence the severity of cyberchondria and its relationship with alexithymia. In sum, these limitations reduce the robustness and generalizability of the findings, underscoring the necessity for future research in more diverse populations and contexts. Moreover, despite the incorporation of both peer-reviewed articles and grey literature in a comprehensive search strategy, the Google Scholar search was limited to the first ten pages due to feasibility constraints, which may have affected the amount of grey literature retrieved.

Future research should address the limitations of the reviewed studies by including different and more representative samples, such as other general population groups, healthcare professionals, and patient populations, including patients with chronic and functional medical conditions and somatic symptoms, as well as by recruiting participants from multiple countries. Future studies should further explore the association between alexithymia and cyberchondria within a broader integrative framework, examining other relevant factors that may influence cyberchondria, such as problematic internet use. The employment of longitudinal and experimental designs would clarify the directionality and causality of the relationship between alexithymia and cyberchondria, as well as the underlying mechanisms involved.

## 5. Conclusions

In conclusion, preliminary evidence suggests that alexithymia may be associated with higher levels of cyberchondria, particularly in relation to difficulties in identifying and describing emotions. However, given the limited and heterogeneous methodological evidence base, these findings should be interpreted with caution until they are confirmed by more robust and longitudinal research. In the current rapidly evolving digital health landscape, future research should also investigate the potential role of generative AI-based tools, such as symptom checkers and conversational chatbots, in shaping cyberchondriac tendencies. Although none of the studies included in this review examined these technologies directly, unrestricted access to online medical information and AI-mediated health consultations may plausibly encourage reassurance-seeking behaviors and maladaptive information-seeking patterns, particularly among individuals with health anxiety and difficulties in emotional processing. Grounded in the Comprehensive Model of Information Seeking, future studies may explore whether interactions with AI contribute to cyberchondria through the interplay between individual vulnerabilities and media-related characteristics [55]. Accordingly, we might speculate that alexithymia represents a relevant personality trait that increases susceptibility to excessive reliance on AI-based health information sources, especially in the context of heightened health-related anxiety. Interventions should extend beyond emotional awareness and regulation to also address the cognitive and informational dynamics of online health consultations. In particular, strategies aimed at reducing information overload and promoting adaptive engagement with AI-based tools may help mitigate cyberchondria and enhance patient–clinician communication in contemporary healthcare settings.

## Figures and Tables

**Table 1:** Characteristics of the included studies.

Authors	Country	Sample	Design	Measure of Cyberchondria	Measure of Alexithymia	Other Relevant Factors	Key Results
Fang et al., 2024 [31]	China	*n* = 7617	Cross-sectional	CSS-12	TAS-20	- Pandemic fear - Psychological distress	- Alexithymia was significantly and positively correlated with cyberchondria. - Alexithymia and psychological distress significantly mediated the relationship between pandemic fear and cyberchondria.
Kalayci et al., 2024 [32]	Turkey	*n* = 190	Cross-sectional	CSS	TAS-20	- Somatosensory amplification	- Alexithymia significantly mediated the relationship between somatosensory amplification and cyberchondria.
Li et al., 2024 [33]	China	*n* = 3977	Cross-sectional	CSS-12	TAS-20	- Pandemic fear	- Network analysis revealed a dense network of predominantly positive associations among pandemic fear, alexithymia, and cyberchondria. - EOT and DIF showed significant negative and positive associations with cyberchondria, respectively.
Sarpdaği et al., 2025 [34]	Turkey	*n* = 415	Cross-sectional	CSS-12	TAS-20	NA	- Both TAS-20 total, DDF, and DIF were significantly and positively correlated with CSS-12 total and its dimensions (EXC, anxiety, REA, and CMP). - EOT showed a significant and positive correlation with the CSS-12 total score, as well as with the REA and CMP scores. - Both CSS-12 total, anxiety, and CMP significantly and positively predicted alexithymia.
Zhou et al., 2022 [35]	China	*n* = 1117	Cross-sectional	CSS	TAS-20	- Stress	- Both TAS-20 total and its dimensions (DIF, DDF, and EOT) were significantly and positively correlated with the CSS total and its dimensions (CMP, DIS, EXC, and REA). - Both TAS-20 total and DIF significantly and positively predicted cyberchondria. - EOT significantly and negatively predicted cyberchondria. - Alexithymia significantly moderated the relationship between stress and cyberchondria.

CMP: Compulsion; CSS: Cyberchondria Severity Scale; DIF: Difficulty Identifying Feelings; DDF: Difficulty Describing Feelings; EOT: Externally Oriented Thinking; EXC: Excessiveness; NA: Not Applicable; REA: Reassurance; TAS-20: Toronto Alexithymia Scale-20.

**Table 2:** Populations and settings.

Study	Population	Setting	*n*	Female (%)	Age (Mean/Range)
Fang et al., 2024 [31]	Nurses	Hospital; post zero-COVID policy	7617	93.4	20–70
Kalayci et al., 2024 [32]	Psoriasis patients and controls	Hospital; Clinical context	190	53.7	18–65
Li et al., 2024 [33]	Nurses	Hospital; post zero-COVID policy	3977	95.2	33.3 ± 7.0
Sarpdaği et al., 2025 [34]	Nurse’s students	University	415	70.1	21.55 ± 1.66
Zhou et al., 2022 [35]	University students	University; COVID-19 period	1117	48.0	19.95 ± 1.32

## Data Availability

No new data were created or analyzed in this study. Data sharing is not applicable to this article.
